# Identifying the features of partner acceptance of arthritis: A qualitative analysis

**DOI:** 10.1080/24740527.2018.1485482

**Published:** 2018-07-23

**Authors:** Kirsten M. Gullickson, Diane L. LaChapelle

**Affiliations:** Department of Psychology, University of New Brunswick, Fredericton, New Brunswick, Canada

**Keywords:** acceptance, partners, arthritis, chronic pain

## Abstract

**Background:**

Patients who are more accepting of their chronic arthritis pain report better physical, mental, and occupational functioning, but acceptance of arthritis from the partner perspective has received little attention in the literature. In fact, no attempts have been made to define partner acceptance of arthritis and no psychometrically validated measure currently exists.

**Aims:**

The aim of this study was to use qualitative research methods to identify the features of partner acceptance of arthritis and examine the similarities and differences between patient and partner acceptance in an arthritis context.

**Methods:**

Twenty-one romantic partners of individuals with arthritis participated in a semistructured interview focusing on their experiences adjusting to their spouse’s arthritis and their general perceptions of the meaning of acceptance. Interview transcripts were coded using thematic analysis.

**Results:**

Partners’ descriptions of acceptance differed slightly across accounts, but the majority of participants agreed that acceptance is part of a positive process of adjusting to arthritis. Six themes that characterize the acceptance process were identified: (1) understanding the nature of arthritis; (2) believing in the patient’s pain experience; (3) living with negative feelings; (4) establishing a new normal; (5) engaging in valued activities; and (6) relationship willingness.

**Conclusions:**

The identified themes share some commonalities with experiential acceptance and patient chronic pain acceptance, although partner acceptance of arthritis also has several unique features. These findings suggest that more research on this distinct construct is merited. Directions for future research on partner acceptance of arthritis are discussed.

## RÉSUMÉ

**Contexte:** Les patients qui acceptent davantage leur douleur arhtirtique chronique font état d’un meilleur fonctionnement physique, mental et professionnel, mais l’acceptation de l’arthtite du point de vue du partenaire a reçu peu d’attention dans la littérature. En fait, aucune tentative pour définir l’;acceptation de l’arthrite par le partenaire n’a été effectuée, et il n’existe actuellement aucune mesure psychométrique valable.

**Objectifs:** Utiliser les méthodes de la recherche qualitative pour cerner les caractéristiques de l’acceptation de l’arthrite par le partenaire et étudier les similitudes et les différences entre l’acceptation par le patient et l’acceptation par le partenaire dans un contexte d’arthrite.

**Méthodes:** Vingt-et-un conjoints de personnes souffrant d’arthrite ont participé à une entrevue semi-structurée portant sur leur expérience d’adaptation à l’arthrite de leur conjoint ou conjointe et sur leur perception de ce que signifie l’acceptation. Les transcriptions d’entrevues ont été codifiées à l’aide d’une analyse thématique.

**Résultats:** Les descriptions de l’acceptation qu’ont fait les partenaires différaient légèrement d’un récit à l’autre, mais la majorité des participants étaient d’accord pour dire que l’acceptation fait partie d’un processus positif d’adaptation à l’arthrite. Six thèmes caractèrisant le processus d’acceptation ont été répertoriés : (1) comprendre de la nature de l’arthrite; (2) croire l’expérience de douleur vécue par le patient; (3) vivre avec des sentiments négatifs; (4) établir une nouvelle normalité; (5) prendre part à des activités que l’on apprécie ; et (6) disposition à entretenir des relations.

**Conclusions:** Les thèmes rèpertoriès ont certains points communs avec l’acceptation expérientielle et l’acceptation de la douleur chronique du patient, bien que l’acceptation de l’arthrite par le partenaire présente également plusieurs caractéristiques uniques. Ces conclusions indiquent que davantage d’études devraient porter sur ce concept distinct. Des pistes de recherche sur l’acceptation de l’arthrite par le partenaire sont abordées.

## Introduction

*Arthritis* is a term used to describe a number of related chronic pain conditions that are characterized by pain, stiffness, swelling of the joints, and fatigue.^1,2^ Arthritis has a pervasive negative impact on patients,^1–4^ and it also affects those closest to the patient, which in most cases is their romantic partner.^5^ Existing literature highlights the widespread consequences of chronic pain conditions such as arthritis for partners: a greater number of reported medical conditions (e.g., high blood pressure, heart disease), poorer sleep quality, greater subjective distress, and an increased incidence of anxiety and depression relative to partners of individuals without chronic pain.^5–10^ Furthermore, partners indicate that they are often required to take on more responsibilities inside the home (e.g., household duties, changing daily routines, offering patients reassurance and assistance), while at the same time having to contribute more outside the home (e.g., increased financial responsibilities, occupational changes) as a result of their spouse’s chronic pain.^8,9,11^ This increased responsibility at home and work has the potential to add to partners’ level of perceived burden and exacerbate the physical and psychological costs of their spouse’s chronic pain.^5,7^ In addition, studies exploring chronic pain’s influence on romantic relationships have demonstrated that patients with chronic pain and their partners report reduced relationship satisfaction, increased sexual dissatisfaction, poor pain-related communication (e.g., criticism/hostility, emotional invalidation, a lack of empathic and validating responses), and decreased social/recreational involvement.^9,11–14^ Research suggests that, in some cases, a lack of relationship satisfaction can lead to dissolution of the relationship (e.g., separation, divorce), which is a significant cost considering the known benefits of being in a supportive romantic relationship for patients with chronic pain.^15–17^

Adjustment to arthritis is a complex and multifaceted process that patients and their romantic partners navigate as individuals and as a couple. Acceptance is one facet of the larger adjustment process that may promote positive partner outcomes. Broadly, experiential acceptance is described as purposefully embracing personal experiences (e.g., thoughts, feelings) as they are, without trying to change or avoid them.^18^ The definition of *acceptance* has also been adapted to make reference to specific physical sensations, such as chronic pain, although the majority of the research has focused on patients rather than partners. Research has suggested that patient chronic pain acceptance consists of two primary components: (1) pain willingness (i.e., refraining from unsuccessful attempts to reduce or avoid pain) and (2) activity engagement (i.e., participation in valued life activities regardless of pain).^19,20^ A third component, chronicity (i.e., acknowledging that a cure for pain is unlikely), was included in early conceptualizations of patient acceptance but was subsequently eliminated because it was found to be unrelated to patient outcomes.^19,20^ Although research supports pain willingness and activity engagement as the two foundational components of patient chronic pain acceptance, studies that have taken a qualitative approach have found that patients do not always describe acceptance in exactly the same way.^20,21^

The association between patient chronic pain acceptance and patient outcomes is complex, but researchers have generally found that patients who are more accepting of their chronic pain condition report better physical (e.g., lower pain severity, decreased physical disability, less need for pain medication, lower levels of medical service utilization), psychological (e.g., less pain-related anxiety, fewer symptoms of depression), and occupational functioning (e.g., greater ability to work).^19,22,23^ Moreover, acceptance and commitment therapy, which includes acceptance as a primary treatment target, is an evidence-based psychological treatment for a variety of physical and mental health problems, including chronic pain and other somatic health conditions, anxiety disorders, depression, and addictions.^24–26^

Given the wide-ranging benefits of acceptance for patients with chronic pain and other individuals from various clinical populations, it can be hypothesized that accepting arthritis also may be advantageous for romantic partners. Unfortunately, partner acceptance represents a significant gap in the existing literature; no attempts have been made to explore the meaning of acceptance from the partner perspective, understand how the partner acceptance process unfolds, or distinguish partner acceptance from patient acceptance in a medical context.

To our knowledge, only two studies to date have considered acceptance from the perspective of partners in a medical context. Both studies attempted to explore the benefits of partner acceptance by adapting existing measures of experiential and patient chronic pain acceptance. As part of a larger study, Pakenham and Samios surveyed partners of individuals with multiple sclerosis and found that those who were more accepting reported fewer symptoms of depression and anxiety, as well as better life and relationship satisfaction.^27^ To measure partner acceptance, the authors used the Acceptance and Action Questionnaire, which was designed to assess acceptance of internal thoughts and feelings, rather than partners’ acceptance in an illness context.^27^ In fact, none of the questionnaire items make reference to multiple sclerosis, illness, or physical sensations. A second study changed the wording of the patient-centered Chronic Pain Acceptance Questionnaire–Revised to reflect partner acceptance and found no significant correlations between partners’ acceptance of their spouses’ vulvovaginal pain and partner outcomes; however, follow-up analyses that controlled for patients’ level of acceptance revealed that higher levels of partner acceptance were associated with lower levels of depression and higher levels of sexual satisfaction.^28^ By adapting the Chronic Pain Acceptance Questionnaire–Revised, these authors made the assumption that patient and partner acceptance of chronic pain are identical constructs, which has not been demonstrated in the literature. It is possible that partner acceptance is a distinct construct that is not adequately quantified by adapted measures of patient acceptance; this may be why Boerner and Rosen found that partner acceptance was not associated with partner anxiety or sexual functioning as they hypothesized.^28^ Although the findings of these two studies provide preliminary evidence of the possible benefits of partner acceptance, neither study attempted to define partner acceptance, nor did they utilize an empirically supported measure of partner acceptance. Until the meaning of partner acceptance is clarified, it will remain unclear whether the results of these two studies are reliable and valid.

The purpose of the current study was to begin to address the aforementioned gaps in the literature by using qualitative research methods to explore the meaning of partner acceptance in an arthritis context. The first goal of the study was to identify the features of partner acceptance of arthritis (i.e., uncover common themes in partners’ arthritis acceptance experiences). The qualitative approach, which gives partners the opportunity to describe their acceptance experiences in their own words, was chosen because we believed that it would result in a richer and more in-depth understanding of partner acceptance. We also expected the results to provide some insight into the acceptance process, although the primary focus of the study was on what partner acceptance is rather than how it unfolds or develops. Given the exploratory nature of the study, no hypotheses about the meaning of partner acceptance were generated prior to the interviews. The second goal of the study was to compare the features of patient and partner chronic pain acceptance to determine whether they are distinct constructs.^19,20^ Although commonalities between partner and patient acceptance were anticipated (e.g., activity engagement), it was also expected that qualities unique to partners’ experiences of acceptance would be identified based on the fact that partners do not experience arthritis firsthand.

## Materials and methods

### Participants

To be included in the study, participants had to be in a cohabitating romantic relationship of at least 12 months with an individual who had been diagnosed with arthritis (a common chronic pain condition). Individuals were excluded from participating if they themselves reported experiencing chronic pain or another serious medical problem (e.g., cancer) in order to ensure that the experiences they described were the result of their spouse’s arthritis. Participants were recruited from across Canada using print advertisements in newsletters and local rheumatologist’s offices, as well as electronic advertisements on national arthritis organization (e.g., Canadian Arthritis Society) and online classified websites. Snowball sampling was also used wherein participants were encouraged to forward the advertisement to any of their relevant contacts.

### Procedure

Ethics approval was obtained from our institutional Research Ethics Board. Advertisements instructed interested participants to contact the primary researcher via e-mail to set up a mutually beneficial time to conduct the interview. Twenty participants were interviewed via telephone and one participant preferred an in-person interview. Following a review of the study information and consent form, verbal consent to participate and audiotape the interview was obtained. To begin the interview, partners verbally responded to a questionnaire that collected relevant demographic (e.g., partner and patient age, education, employment status), health (e.g., diagnosis, time since diagnosis), and relationship information (e.g., marital status, relationship duration). Partners subsequently took part in a semistructured interview (see Table 1 for the interview schedule). In short, partners were asked to describe how their spouse’s arthritis impacted them and describe what it means to accept those impacts. Given the exploratory nature of the current study, the research questions were as broad as possible, but the word *accept* was included in one of the questions to ensure that interviews focused on the intended topic. Participants were prompted to provide further detail, offer additional examples, or clarify their responses when necessary. Although the prompts were based on existing literature (e.g., arthritis’s impacts on partners, components of patient chronic pain acceptance), the primary interview questions were not theoretically derived. All interviews were conducted by the first author and lasted an average of 60 min (range 35 to 120 min). The decision to discontinue recruitment was made by the interviewer at the point of saturation (i.e., the point at which no novel information was being elicited from the interviews).

**Table 1.** Semistructured interview schedule.

1. How has your spouse’s arthritis affected you as an individual, a couple, and a family?
● What challenges have you faced as a result of your spouse’s arthritis?
● Are there any positive implications of arthritis you can identify?
2. What does it mean to accept your spouse’s arthritis?
● In what ways have you adjusted to the impacts of arthritis you mentioned previously?
● Do you feel like you have come to terms with the fact that your spouse’s arthritis is not going away and that the symptoms are chronic?
3. Is there anything you would like to share that we haven’t covered already?

*Note*: Research questions are in numbered order. Potential probes for each question are listed in bullet form.

### Analysis

Audiotapes were transcribed verbatim and identifying details were removed to ensure anonymity and confidentiality. Once the transcripts were verified for accuracy by the first author, the content was coded in an iterative fashion using thematic analysis.^29^ Thematic analysis was chosen because it is a flexible and accessible approach that is commonly used in psychological research to answer a variety of research questions. Furthermore, it allowed us to take an inductive thematic approach, wherein the data were coded without the use of a preexisting coding frame; this meant that rather than searching for certain pre-identified themes within the transcripts, we let the content of the interviews dictate the codes and themes. Although an inductive approach is not theory driven, it is important to note that “data are not coded in an epistemological vacuum” (p. 84); therefore, it is possible that our interpretation of the data was inadvertently influenced by our prior knowledge of acceptance theory.^29^

The first step in the analytic process was to systematically analyze the entire data set to generate initial codes (i.e., single ideas present in the data). When generating codes, we focused primarily on the surface meaning of the partners’ accounts and content that was explicitly stated by the partners (i.e., semantic content and theme development). During this phase, the researchers met regularly to discuss codes identified in the transcripts. Subsequently, an essentialist/realist approach was used when the codes were grouped into potential themes (i.e., common, recurring concepts) that we believe represent the features of partner acceptance of arthritis. Next, the primary researcher completed a secondary analysis of all transcripts to refine the specifics of each code and theme (e.g., search for missed codes, correct miscoded content, consider alternative code groupings). Finally, a different researcher, who was familiar with but not directly involved in the study, coded 10% of the transcripts to ensure that no major codes or themes had been missed or misinterpreted in the analysis. If discrepancies did arise, they were discussed at length and, if necessary, transcripts were re-examined to recode any such content.

## Results

### Demographics

In total, 21 partners of individuals with arthritis participated in the study. The mixed-gender (38% male) partner sample was mostly Caucasian, highly educated (i.e., at least some university), and employed on a full-time basis (see Table 2). Partners reported being in committed (married = 16; common-law = 5), heterosexual relationships for an average of 15.3 years (SD = 15.4 years; range = 1–49 years).

**Table 2.** Demographic and health information for partners and patients.^a^

	Partner	Patient
**Gender**		
Male	8 (38.1%)	13 (61.9%)
Female	13 (61.9%)	8 (38.1%)
**Age (years)**		
Range	21–68	19–67
Mean (SD)	42.7 (12.0)	43.3 (11.8)
**Ethnicity**		
Caucasian	19 (90.5%)	17 (81.0%)
Other^b^	2 (9.5%)	4 (19.0%)
**Education**		
Some university or more	19 (90.5%)	17 (81.0%)
High school degree	2 (9.5%)	4 (19.0%)
**Employment status**		
Full-time	13 (61.9%)	11 (52.4%)
Part-time	3 (14.3%)	1 (4.8%)
Student	2 (9.5%)	2 (9.5%)
Retired	2 (9.5%)	3 (14.3%)
Unemployed (disability)	—	2 (9.5%)
Unemployed (other)	1 (4.8%)	2 (9.5%)
**Arthritis type**		
Rheumatoid arthritis	—	10 (47.6%)
Osteoarthritis	—	3 (14.3%)
Ankylosing spondylitis	—	2 (9.5%)
Psoriatic arthritis	—	2 (9.5%)
Other^c^	—	4 (19.0%)
**Time since diagnosis (years)**		
Range	—	0.5–37
Mean (SD)	—	9.4 (9.8)
**Timing of arthritis onset**		
Pre relationship formation	—	10 (47.6%)
Post relationship formation	—	11 (52.4%)

^a^All information was provided by partners.

^b^Other: Asian, African, West Indian.

^c^Other: Juvenile arthritis, unknown/unable to remember.

### Impact of arthritis on partners

Although a full review of the impacts of arthritis described by partners is beyond the scope of this article, it is important to briefly summarize what the partners reported, because partner acceptance is likely linked to these experiences. Overall, despite the heterogeneity of the sample with regard to type of arthritis and life stage (e.g., age, employment status), partners consistently reported that their spouses’ arthritis had widespread negative implications. At the individual level, arthritis was detrimental to partners’ physical (e.g., stress levels, sleep quality, diet/exercise habits) and emotional (e.g., frustration, sadness, helplessness, guilt, worry) well-being. At the relationship level, arthritis had a negative impact on household management, parenting, occupational functioning, financial security, and social/recreational involvement. Numerous partners also reported that arthritis had been detrimental to their relationship satisfaction (e.g., poor communication, lack of intimacy, physical/emotional changes in the patient).

### Self-reported acceptance

Partners used the term acceptance and other synonymous phrases, such as “learning to live with,” “coming to terms with,” “dealing with,” “carrying on despite,” and “getting used to” arthritis, interchangeably to describe a positive process of adjustment to arthritis. A small number of partners perceived the term acceptance to have a negative connotation, believing it to be analogous with resignation, “giving up,” or “being defeated.” Interestingly, when those individuals were encouraged to use their preferred language, they used the synonymous phrases listed above to describe the same adaptive adjustment process as those who embraced the term acceptance. This finding indicated that though a select number of partners disliked using the word acceptance, their descriptions of their experiences were similar regardless of their preferred terminology.

Although partners were not explicitly asked about their perceived level of acceptance, the majority of partners self-identified as being accepting of their spouse’s arthritis at some point during the interview. When explaining her perspective on acceptance, one partner said, “It took me a while to accept [his arthritis] … to accept that he didn’t ask for [arthritis], it’s not his fault, but now I have” (female, married 6 years to a patient diagnosed with rheumatoid arthritis 2 years ago). Acknowledging acceptance was possible even in the face of a changing illness, as another partner indicated, “I have accepted that I will be with someone that will be in pain 24/7 for the rest of our lives … even if there are hurdles, I accept that lifestyle that we have” (female, married 4 years to a patient diagnosed with rheumatoid arthritis 19 years ago).

In contrast to those who self-identified as accepting, several partners described their continued difficulty adjusting to their spouse’s arthritis and expressed their lack of acceptance. One partner’s ambivalence about acceptance was evident when she said, “Maybe I should try to be more accepting … but when you’re used to something and it all of a sudden changes, it’s hard to be accepting … especially because, like I said, I’m burnt out, I’m frustrated” (female, common-law marriage of 22 years to a patient diagnosed with arthritis 2 years ago). Moreover, whereas some partners were optimistic that they would one day learn to accept their spouse’s arthritis, others questioned whether they would ever be accepting. For example, one partner reported, “I know that I haven’t accepted it yet. … I’m past the denial stage, because I know that he’s had this for a while now, but I don’t know how I can ever accept it” (female, married 5 years to a patient diagnosed with rheumatoid arthritis 10 years ago).

### Acceptance themes

Partners struggled to provide a comprehensive, concrete definition of acceptance and some had diverse views on the meaning of acceptance, although they were able to describe their experiences adjusting to the impacts of their spouse’s arthritis and share their general perceptions of the acceptance process. Overall, the majority of partners perceived acceptance to be a positive process of adjustment that occurs over time. Although partners were not explicitly asked about the process of acceptance, most indicated that acceptance developed over the course of several years. Partners described acceptance as being dynamic in nature, because the evolution of the patient’s arthritis required them to constantly readjust. From the descriptions provided by partners, six themes that are thought to represent the features of partner acceptance were identified: (1) understanding the nature of arthritis; (2) believing in the patient’s pain experience; (3) living with negative feelings; (4) establishing a new normal; (5) engaging in valued activities; and (6) relationship willingness. The themes reported by partners did not appear to differ based on age, gender, or the patient’s type of arthritis, but the accounts of partners who had lived with their spouse’s arthritis for longer tended to include a greater number of acceptance themes. In general, the number of themes identified in the partners’ narratives varied, with some partners describing most or all of the themes and others describing only one or two of the themes. Each theme is described below and illustrated with quotes.

#### Theme 1: Understanding the nature of arthritis

According to partners, acceptance begins with learning to understand that arthritis is a chronic condition for which there is no cure despite the best available treatments. When one partner was describing his definition of acceptance, he explained, “To accept it … to me I think it is coming to terms with [arthritis] and understanding what it means and what is involved” (male, married 11 years to a patient diagnosed with rheumatoid arthritis 5 years ago).

Numerous partners indicated that, once arthritis became a part of their lives, the initial step in the adjustment process involved learning about the illness. Specifically, partners obtained information about arthritis symptoms, treatment, and prognosis from medical professionals, the patients themselves, and print resources (e.g., the Internet, books). Subsequently, partners began to assimilate their newfound knowledge and recognize that the patient would likely never be 100% pain free, even with the most effective treatment options being utilized. They also began to recognize the futility of attempting to control their spouse’s pain. One partner articulated this point when she explained:

To me, [being accepting] means to just accept that fact that [arthritis] is here to stay … he can’t just take medication and it’s going to be gone. It’s going to be something we have to deal with and it’s going to get worse and just accept the fact that it’s going to affect our lives forever. (female, married 6 years to a patient diagnosed with rheumatoid arthritis 2 years ago)

In some cases, partners described coming to terms with the progressive nature of the patient’s condition, which meant acknowledging that the patient’s symptoms, and thus arthritis’s impact on their lives as partners, might increase over time. When describing his outlook on the future, one partner stated, “I know it’s going to get worse … it can stabilize somewhat [with medication], but it’s never going to heal … we both know that by now” (male, married 49 years to a patient diagnosed with rheumatoid arthritis 20 years ago).

#### Theme 2: Believing in the patient’s pain experience

Acceptance also involves gradually learning to trust that the patient’s pain is as persistent and severe as reported. Some partners acknowledged that they occasionally questioned whether their spouse’s pain was exaggerated or fabricated. Although she had overcome her doubts, one partner acknowledged questioning her partner’s experience in the past when she said, “Until I realized how bad it was and how serious it was, I would get frustrated. … I guess it took me a while to realize that he wasn’t making it up and he was in pain and it was real” (female, married 6 years to a patient diagnosed with rheumatoid arthritis 2 years ago).

Most partners indicated that their belief in the authenticity, severity, and chronicity of their spouse’s pain was solidified over time as they learned to identify when the patient was in pain. They did so by attending to their spouse’s verbal (e.g., groans, sighs) and nonverbal cues (e.g., limping, grimacing, mood) rather than relying exclusively on their spouse’s reports. One partner explained that he could tell that his spouse was in pain just by looking at her: “You just see the black under her eyes … she’s in pain” (male, married 38 years to a patient diagnosed with psoriatic arthritis 37 years ago). Another partner described the numerous ways by which she could tell her husband was in pain by saying, “I see it in the way he walks and the way he moves … he’s not able to turn his head. Even his irritability, it’s obvious to me. When he’s irritable I can put two and two together [he’s in pain]” (female, married 4 years to a patient diagnosed with ankylosing spondylitis 2.5 years ago).

#### Theme 3: Living with negative feelings

Many partners associated acceptance with the ability to live life without ruminating on negative feelings related to their spouse’s arthritis. Partners described experiencing frustration, sadness, guilt, helplessness, and worry as a result of arthritis, but noted that dwelling on their negative feelings and fighting something they had little power to change was both unproductive and exhausting. In one interview, the partner acknowledged her frustrations with her spouse’s arthritis but explained how she gradually realized that focusing on the consequences of his arthritis was unhelpful: “I’ve just been frustrated [with his arthritis], negative all the time. And you know it takes the toll on you. … I’ve started to realize, you know, it’s not good, health wise, to be frustrated and I just had to let it go” (female, common-law marriage of 1 year to a patient diagnosed with osteoarthritis 3 years ago). Rather than focusing her attention on things that are out of her control, another partner described her efforts to redirect her energy toward things within her control: “There’s no point in dwelling on stuff we can’t really change. … I have come to realize a lot more … I can’t control that, but I can control me and my reactions to it and how I deal with it” (female, married 6 years to a patient diagnosed with rheumatoid arthritis 9 years ago).

Although partners found it extremely challenging at times to move past their negative feelings, they described numerous strategies that they adopted to help them. A number of partners reported that being proactive and planning for the future helped ease their worries. Some described engaging in cognitive reappraisal of their negative thoughts, whereas others noted that being mindful (e.g., living in the moment, focusing on the present) was a useful strategy for shifting their attention away from negative emotions. One partner articulated his mindful approach to his spouse’s arthritis when he said, “We take it one day at a time and I don’t over analyze things. I just go with the flow” (male, common-law marriage of 7 years to a patient diagnosed with osteoarthritis 13 years ago).

Importantly, partners noted that living with negative feelings did not necessarily mean being enthusiastic about having arthritis as a part of their lives, nor did it mean being resentfully resigned to a life including arthritis; rather, acceptance involved making peace with their spouse’s arthritis. One partner described this perspective by saying, “To me I think [acceptance] is coming to terms with [arthritis] and being, I guess, generally okay with it … not necessarily loving it, but understanding it” (male, married 11 years to a patient diagnosed with rheumatoid arthritis 5 years ago). Another partner echoed these sentiments: “I don’t think acceptance means you have to be positive about it, but I think you have to be okay with it … not negative or miserable” (female, common-law marriage of 1 year to a patient diagnosed with osteoarthritis 3 years ago).

Some partners noted that shifting their attention away from the negative allowed them to focus on the positive aspects of their lives. Several partners were able to identify at least one positive outcome to result from arthritis. For example, one partner explained how his spouse’s arthritis had helped him grow as an individual:

I think [my wife’s arthritis] has certainly made me a lot more empathetic and a lot more aware of people with [invisible] disabilities. I mean everywhere you can see the person in the wheelchair, but you can’t always see the person who has arthritis or the person who has multiple sclerosis. (Male, married 6 years to a patient diagnosed with rheumatoid arthritis 9 years ago)

Another partner described how dealing with her spouse’s arthritis had strengthened their romantic relationship:

[Arthritis] has maybe even encouraged us to be closer emotionally. You know, I have to be a little more in tune with how he’s doing. … We spend a lot of time together because he’s been unwell. … It helps you grow stronger as a couple on an emotional level. (Female, married 20 years to a patient diagnosed with unspecified arthritis 3 years ago)

#### Theme 4: Establishing a new normal

Partners repeatedly indicated that accepting arthritis involves being open to making necessary lifestyle adjustments in order to accommodate their spouse’s chronic pain. According to partners, this process was gradually initiated when they came to the realization that they could no longer maintain their pre-arthritis lifestyle. One partner acknowledged the various ways in which he and his wife would have to adjust when he said, “I know that we need to make some changes with regard to how our life is currently run and how that will affect us, you know, relationship, mentally, physically, et cetera” (male, married 11 years to a patient diagnosed with rheumatoid arthritis 5 years ago). Consequently, partners indicated they eventually redefined normal based on the limitations imposed by the patient’s arthritis. One accepting partner articulated his perspective on the new normal by saying, “We live in a limited environment. I live in a limited environment because of her so I’ve accepted that, so it became the normal” (male, married 38 years to a patient diagnosed with psoriatic arthritis 37 years ago).

For the majority of partners, establishing a new normal meant shifting their way of thinking about themselves, the patient, and the relationship in the context of arthritis. One partner described her willingness to make adjustments by saying, “Yeah me, personally, I had to make changes and it’s because of him, but it’s nothing bad, it’s just me deciding, yeah, I’ll make those sacrifices or those changes” (female, married 4 years to a patient diagnosed with rheumatoid arthritis 19 years ago).

Numerous partners described how they had adjusted their expectations as a result of arthritis. For example, in describing her outlook on the household division of labor, one partner noted, “[It’s] not realistic to expect him to be able to contribute the same way that I contribute in doing stuff around the house” (female, married 6 years to a patient diagnosed with rheumatoid arthritis 9 years ago). Similarly, another partner described negotiating parenting roles to accommodate her husband’s arthritis: “We registered the kids again for gymnastics this year and you know when I registered them I knew that this was my activity. So he comes and he watches, but I don’t have the expectation that he’s going to assist” (female, married 6 years to a patient diagnosed with rheumatoid arthritis 2 years ago).

In addition, partners described making practical adjustments to their day-to-day routines within and outside of the home to ease the burden of arthritis. One partner described how she adjusted household chores as a result of her spouse’s arthritis: “I’ll do stuff that requires kneeling, like the toilet or the bathtub or stuff like that, he’ll do stuff that needs to stand up” (female, married 4 years to a patient diagnosed with rheumatoid arthritis 19 years ago). Another explained how she had changed her work schedule to accommodate her spouse’s arthritis:

I’m trying to change my hours so that I can be home with [our daughter] more, so that I can be home just to make sure that [my husband] is okay. I’m just trying to do small little things like that, that can hopefully make a difference … that can hopefully make things easier stress wise for both of us. (Female, married 24 years to a patient diagnosed with rheumatoid arthritis 2 years ago)

A third partner described how he and his spouse had modified their leisure activities as a result of arthritis:

You can still do a lot of activities, you just have to modify them a bit. … Like I said, going for a five kilometer walk, which maybe previously it would have been a jog, and then got knocked down to just a fast walk … there are other days where you’re going to stop every kilometer and sit down on a park bench. … (Male, married 3 years to a patient diagnosed with ankylosing spondylitis 10 years ago)

Partners explained that as they became more comfortable in their new behavioral patterns, their confidence in their ability to handle arthritis-related hurdles increased. Partners noted that their sense of normality continued to evolve with the patient’s arthritis, such that they had to be willing to continue readjusting to accommodate the progression of the patient’s arthritis. One partner described his outlook on the future by saying, “[When something new comes up] everything stops for a short period of time, until we make our adjustments and then we start to move forward again” (male, married 11 years to a patient diagnosed with rheumatoid arthritis 5 years ago).

#### Theme 5: Engaging in valued activities

Partners indicated that acceptance involves finding meaning and fulfillment through valued activities as a couple and an individual despite arthritis. A number of partners described positive effects on the relationship when they continued to engage in mutually enjoyable recreational and social activities notwithstanding the limitations imposed by the patient’s arthritis. One partner demonstrated the value he placed on engaging in activities as a couple by saying, “I think it doesn’t matter what we’re doing, as long as we’re doing things together” (male, common-law marriage of 1 year to a patient diagnosed with juvenile idiopathic arthritis 11 years ago).

Partners stressed the importance of choosing activities the couple could engage in regardless of the patient’s level of pain, because they acknowledged complete symptom relief was unlikely. In some cases, partners and their spouses were able to stay involved in the same activities they had enjoyed prior to arthritis, whereas others had modified their pre-arthritis activities or found new activities. When describing the various activities he and his wife engaged in as a couple despite her pain, one partner explained:

We can go downtown, we can go shopping, we can do things, you know. We can take her camera and we go for a drive let her take pictures everywhere, you know she likes that, I think that, I love that. (Male, married 38 years to a patient diagnosed with psoriatic arthritis 37 years ago)

Another partner acknowledged the effort he and his wife put in to find a mutually enjoyable activity that was not limited by her arthritis: “We went to try to figure out what could we do that would be fun and do together. The sailing came up and we’ve just fallen in love with this thing and we just love the whole experience” (male, married 6 years to a patient diagnosed with rheumatoid arthritis 6.5 years ago).

In addition to staying active as a couple, partners described the importance of maintaining their personal happiness by engaging in valued activities independently or with family and friends, even if the patient was unable to participate. One partner explained her decision not to let her husband’s pain limit her pursuit of personally valued activities when she said:

If I want to do something and he doesn’t feel like it I just have to go and do it on my own if I want to. I can’t wait around for him to want to go do stuff because it might be days before he feels like he wants to do whatever it is. So I just go on my own. (Female, married 6 years to a patient diagnosed with rheumatoid arthritis 9 years ago)

Another partner expressed a similar sentiment and noted that her husband was supportive of her engaging in valued activities independently:

We choose not to let [his arthritis] limit the rest of us. So if he doesn’t want to ski, we are not sitting home or not doing it because he does not want to or can’t. We are going anyway and he is very encouraging that we do. (Female, married 14 years to a patient diagnosed with psoriatic arthritis 30 years ago)

Although participating in enjoyable activities as an individual sometimes resulted in feelings of guilt about leaving the patient behind, partners stressed the value of maintaining autonomy and living life for more than just caregiving or housekeeping. This helped partners preserve a sense of fulfillment despite their spouses’ arthritis.

#### Theme 6: Relationship willingness

Throughout their accounts, many partners implied that being accepting of their spouse’s arthritis involved a willingness to commit themselves to an arthritis-affected relationship. More specifically, most partners expressed their intention to stay in their current relationship despite the challenges posed by their spouse’s arthritis. Statements made by these partners conveyed their commitment to their spouse and their openness to tackling any challenge arthritis may have posed. When describing his perspective on staying in the relationship, one partner said, “I’ve committed myself to our marriage. I’m in it for the long haul [even though she has arthritis]. It’s part of the marriage vows, through sickness and health. I feel very strongly about that” (male, married 13 years to a patient diagnosed with osteoarthritis 12 years ago).

In contrast, a few partners seemed uncertain about whether they were willing to remain in their current relationship because of their spouse’s arthritis. These partners were considering the possibility of terminating their relationship so they would no longer have to deal with their spouse’s arthritis and its impacts. One partner’s ambivalence was evident when she stated:

I’m thinking of [leaving him]. … Which is sad because you invest so many years with somebody, but at the end of the day if you’re not happy, then what? Do you live like this [with his arthritis] for the rest of your life? Because I can’t see it happening honestly. (Female, common-law marriage of 22 years to a patient diagnosed with arthritis 2 years ago)

## Discussion

To our knowledge, the current study is the first attempt to determine the meaning of partner acceptance in an arthritis context. In order to identify the features of partner acceptance of arthritis, romantic partners were asked to describe the impact of their spouses’ arthritis on their lives and explain what acceptance of arthritis meant to them. Consistent with existing literature, partners emphasized the pervasive impact of their spouse’s arthritis at the individual (e.g., physical and emotional well-being) and relationship (e.g., household management, financial security, relationship satisfaction) levels.^5–17^ Although the implications of arthritis and other chronic pain conditions for partners has been the subject of past research, we are unaware of any previous research that has explored the comprehensive impact of arthritis in an acceptance context. For the purposes of the present study, learning about the consequences of arthritis for partners provided insight into the acceptance process by clarifying the various ways in which partners have adapted to their spouse’s chronic pain.

When asked to define acceptance of arthritis from their perspective, partners struggled to provide a comprehensive, concrete definition, which is likely due to the abstract nature of the construct. Alternatively, it may have been challenging for those who had not yet experienced acceptance to describe its features. Nevertheless, partners described their unique experiences and provided diverse insights into the meaning of partner acceptance. Not every partner defined acceptance using the same terms or described their experiences in the same way, which is consistent with findings from another qualitative study in which patients were asked to identify the features of chronic pain acceptance.^20^ Nevertheless, partners generally agreed that acceptance of arthritis is an adaptive process that unfolds over the course of several years. Partners also indicated that acceptance progresses in a dynamic and nonlinear fashion, because fluctuations in patients’ symptoms require partners to re-adjust continuously. Together, these finding suggest that acceptance is a lengthy, ongoing part of the larger adjustment process. Variability in the course of partner acceptance may be the result of a number of partner, patient, and relationship factors, such as the partner’s prior knowledge of and experience with arthritis, the severity and chronicity of the patient’s pain, the patient’s level of acceptance, and the premorbid quality of the relationship (e.g., relationship satisfaction); however, more research is needed to test these hypotheses.

Examination of the content of the partner accounts led to the identification of six themes that we believe represent the features of the partner acceptance: (1) understanding the nature of arthritis; (2) believing in the patient’s pain experience; (3) living with negative feelings; (4) establishing a new normal; (5) engaging in valued activities; and (6) relationship willingness. The themes described by partners did not appear to differ based on gender, suggesting that acceptance means the same thing to male and female partners. This is consistent with existing patient research, which has not reported any significant gender differences in acceptance.^18–20,22–25^ Notably, the accounts of partners who had lived with their spouse’s arthritis for longer tended to include a greater number of acceptance themes, which supports the idea that partners become more accepting of their spouse’s arthritis over time.

The identified themes were compared to the components of experiential and patient chronic pain acceptance to determine whether partner acceptance of arthritis is distinct from existing conceptualizations of acceptance. The comparison revealed that partner acceptance of arthritis shares several features with both experiential and patient chronic pain acceptance. Understanding the nature of arthritis (theme 1) has commonalities with the pain willingness component of patient chronic pain acceptance (i.e., refraining from unsuccessful attempts to reduce or avoid pain).^18,19^ It also is similar to McCracken and colleagues’ chronicity component (i.e., recognizing the chronicity of pain and giving up the search for the cure), which emerged during the original factor analysis but was subsequently eliminated because it was found to be unrelated to patient outcomes (e.g., pain intensity, physical and psychosocial disability, work status).^18,19^ Living with negative feelings (theme 3) does not correspond directly with the features of patient chronic pain acceptance, but it shares similarities with experiential acceptance (i.e., purposefully embracing feelings, thoughts, and sensations as they are without trying to change or avoid them), a more general form of acceptance.^22^ Finally, engaging in valued activities (theme 5) is consistent with the activity engagement component of patient chronic pain acceptance (i.e., behaving in a manner consistent with personal values regardless of pain).^18,19^ In summary, despite the fact that patients and partners experience arthritis from different perspectives (i.e., firsthand vs. indirectly), acceptance for both parties involves limiting attempts to eliminate or control pain, learning to live with the functional and emotional impacts of arthritis on daily life, and making a purposeful effort to move forward with life despite pain. These shared features could speak to the potential benefits of partner acceptance, given that existing research has highlighted their relationship to positive patient outcomes.^19,22,23^

The three remaining themes do not correspond with the components of experiential or patient chronic pain acceptance, which suggests that they may represent unique features of partner acceptance. Relationship willingness (theme 6) is the theme that most clearly distinguishes partner from patient acceptance: Partners can choose to terminate the relationship in order to escape the effects of arthritis—an option that patients do not have. Believing in the patient’s pain experience (theme 2) is also a distinct feature of partner acceptance. Given that there is no direct objective measure of pain experiences, partners face the unique challenge of having to trust in the patient’s communicated pain experience. Lastly, although establishing a new normal (theme 4) has no obvious patient acceptance counterpart, we believe that it is a process that both patients and partners go through when adjusting to chronic pain. The fact that this theme is not included in current conceptualizations of patient acceptance may suggest that it is not a component of acceptance per se but perhaps precedes or parallels the development of acceptance as part of the larger adjustment process.

Results of this study clearly suggest that partner and patient acceptance are similar but not identical constructs. As such, it cannot be assumed that partners will progress through the process of acceptance in the same fashion as patients or experience the same benefits of acceptance that have been identified for patients. Furthermore, given that there are several unique components to partners’ experiences with acceptance, using adapted measures of patient acceptance to assess partner acceptance is likely to miss important aspects of their experience. Consequently, the results of the two existing studies on partner acceptance that utilized existing acceptance measures are of limited value.^27,28^ Additional research is needed to further refine the definition of partner acceptance, expand our longitudinal understanding about the development of partner acceptance, and create a psychometrically validated measure that can be used to evaluate the benefits and correlates of partner acceptance in research and clinical practice.

### Clinical relevance

Although the exploratory and qualitative nature of this study preclude us from drawing definitive conclusions about the benefits of partner acceptance, analysis of the interview content suggests that there is a positive relationship between partner acceptance and partner outcomes. Specifically, partners who identified with most or all of the acceptance themes appeared to be experiencing more positive outcomes compared to those who described fewer themes. For example, they tended to report less emotional distress, better daily functioning, more social/recreational involvement, and greater relationship satisfaction. In contrast, partners who acknowledged their lack of acceptance indicated that they were experiencing significant personal and relationship distress as a result of their spouse’s arthritis. If future research confirms that partner acceptance predicts better partner outcomes, then facilitating partner acceptance could have significant benefits for both partners and patients. For instance, given that partners who report greater relationship satisfaction tend to provide better quality support (i.e., more adaptive responses to the patient’s pain), cultivating partner acceptance could indirectly contribute to improved patient outcomes.^30^

It should be noted that the majority of the accepting partners in the current study learned to accept their spouse’s arthritis without professional help, suggesting that for many partners acceptance develops naturally over time; however, a subset of partners may come to clinical attention when their personal well-being and relationship quality have deteriorated, perhaps to the point where they are considering terminating the relationship. Based on the findings of the present study, it appears that a combination of acceptance-based therapy (e.g., mindfulness, values identification, committed action) and skills focused cognitive behavioral therapy (e.g., communication skills, improved understanding of arthritis/chronic pain) could help partners improve their personal and relationship well-being; however, given the preliminary nature of our understanding of partner acceptance, additional research is needed to confirm the association and directionality of the relationship between partner acceptance and partner outcomes, as well as to investigate the benefits of clinical intervention.

Lastly, the results of the current study highlight the importance of clarifying partners’ preferred terminology when discussing acceptance in a clinical setting. Although the vast majority of partners perceived acceptance to be a positive process, a few partners perceived the term acceptance to have a negative connotation, believing it to be akin to giving up or being defeated. This finding is similar to what has been found for patients with arthritis and fibromyalgia and highlights the importance of determining partners’ preferred terminology or clarifying the meaning of the term acceptance.^21^ Furthermore, researchers working to develop ways of assessing partner acceptance should consider using alternative terminology (e.g., “coming to terms with”) or clarify what is meant by the term acceptance to ensure that the chosen terminology does not bias the partners’ responses.

### Limitations

Despite filling an important gap in the literature, the present study had several limitations. First, this preliminary study explored partner acceptance of arthritis independent from patient chronic pain acceptance. By focusing exclusively on partners’ acceptance experiences without considering the influence of patient acceptance, it is possible that we have understated the complexity of partner acceptance of arthritis. In reality, arthritis is a dyadic stressor that stimulates individual and dyadic adjustment efforts on the part of the patient and partner; thus, it is reasonable to assume that there is a reciprocal or bidirectional relationship between patient and partner acceptance, such that partners are more likely to be accepting of arthritis if the patient is also accepting and vice versa. Future research exploring the development and course of acceptance would benefit from a dyadic approach that considers the interaction between patient and partner.

Second, it is likely that the partners who volunteered for the study were more accepting and less distressed than those who chose not to participate or those who did not meet the inclusion criteria for the study. For example, there is a strong possibility that the partners who participated in the study were generally more satisfied with their relationships; thus, the results of the current study are likely more reflective of the experiences of accepting partners. Furthermore, by excluding individuals who were previously, but not currently, in an arthritis-affected relationship, we may have missed out on the accounts of individuals who terminated their relationship as a result of their spouse’s arthritis. If we had included those individuals we may have obtained richer data from partners who were unwilling to accept arthritis. Although we made a concerted effort to recruit a heterogeneous sample of partners (e.g., diverse in terms of gender, length of relationship, and patient diagnosis), the results of the current study are limited by this sampling bias.

Third, because our sample was exclusively Canadian and primarily Caucasian, it is unclear to what extent these findings are representative of partners from other countries or of other ethnicities. Differences in health care availability across countries and cultural variations in lifestyle (e.g., division of household labor, relationship expectations) may influence the degree to which partners are affected by arthritis and thus their acceptance experiences. Despite this limitation, it is likely that the findings of the present study are at the very least representative of Caucasians from North America. Additional research may be needed to elucidate geographical and/or cultural differences in partner acceptance.

Fourth, we cannot confirm the generalizability of the study results to partners affected by other chronic pain conditions because inclusion was restricted to partners of individuals with arthritis. It is possible that certain illness characteristics, such as treatment availability and effectiveness, illness progression, and diagnostic uncertainty, may subtly affect partners’ acceptance experiences. For example, it is unclear how the diagnostic ambiguity of fibromyalgia impacts how partners understand the nature of the illness or their trust in their spouse’s pain experience. We hypothesize that the themes identified in this study are representative of partners with spouses experiencing various chronic pain conditions given that pain and fatigue are relatively consistent symptoms across chronic pain conditions; however, more research is needed.

Finally, it is possible the partners who participated in this study intentionally or unintentionally engaged in socially desirable responding. For instance, the vast majority of partners interviewed identified themselves as accepting of their spouse’s arthritis, yet several made contradictory statements elsewhere in the interview. This discrepancy may have been a result of partners feeling internalized pressure to fulfill the supportive partner role (i.e., a supportive partner would be accepting). Despite this potential social desirability bias, partners provided detailed descriptions of their acceptance experiences and did not present an overly positive picture of the impact that arthritis had on their lives; in fact, they described at length the challenges they faced coming to terms with their spouses’ arthritis.

### Future directions

Although this exploratory research serves as a valuable starting point in the study of partner acceptance of arthritis, there is still much to learn. It is our hope that this study will stimulate a wealth of additional qualitative and quantitative research. Subsequent qualitative research is required to dig deeper into each of the identified partner acceptance themes and further refine the meaning of partner acceptance. For example, more research is needed to confirm that these themes are representative of partners’ acceptance experiences (i.e., the themes should be presented to partners so that they can provide feedback). Additional qualitative research also is needed to understand the course of partner acceptance. For instance, partners could be asked to identify critical junctures in the development of acceptance, as well as barriers and facilitators of the acceptance process. Furthermore, partners should be given the opportunity to explain how acceptance has impacted their lives at the individual, relationship, and family levels. Additionally, future research should explore how acceptance relates to other facets of the broader adjustment process (e.g., individual and couple identity reformulation, individual and dyadic coping efforts).

Once more is known about the meaning and process of partner acceptance, a self-report measure can be developed and psychometrically validated. Exploratory factor analysis will help reveal the key factors that underlie partner acceptance of arthritis and clarify the relationships among the factors. For example, we hypothesize that partner acceptance has a hierarchical factor structure, wherein relationship willingness is a higher-order factor that subsumes the other five themes. A psychometrically sound measure could subsequently be used in future quantitative research to elucidate the relationship between partner acceptance of arthritis and partner, patient, and relationship outcomes. Specifically, researchers could examine what aspects of partner acceptance are the strongest predictors of partner outcomes, what coping strategies are associated with partner acceptance, how partner acceptance influences the quantity and quality of support provided to the patient, and how demographic, illness, and contextual variables affect the partner acceptance process. Future research could also tease apart the relationship between partner and patient chronic pain acceptance processes. Finally, longitudinal research can use a partner acceptance questionnaire to measure how acceptance unfolds over time or monitor treatment progress.

Lastly, future research can evaluate whether cultivating partner acceptance is an effective intervention strategy to improve partner, patient, and relationship outcomes (e.g., partner and patient well-being, relationship satisfaction). Researchers can then compare the effectiveness of individual and couples therapy to determine the most appropriate way to treat patients and partners who are struggling to adjust to arthritis.
